# Glycosylation on Hemagglutinin Affects the Virulence and Pathogenicity of Pandemic H1N1/2009 Influenza A Virus in Mice

**DOI:** 10.1371/journal.pone.0061397

**Published:** 2013-04-24

**Authors:** Yan Zhang, Jiping Zhu, Yongtao Li, Konrad C. Bradley, Jiyue Cao, Huanchun Chen, Meilin Jin, Hongbo Zhou

**Affiliations:** 1 State Key Laboratory of Agricultural Microbiology, College of Veterinary Medicine, Huazhong Agricultural University, Wuhan, People's Republic of China; 2 Department of Microbiology and Immunology, Emory University School of Medicine, O. Wayne Rollins Research Center, Atlanta, Georgia, United States of America; Alberta Provincial Laboratory for Public Health/University of Alberta, Canada

## Abstract

The two glycosylation sites (Asn142 and Asn177) were observed in the HA of most human seasonal influenza A/H1N1 viruses, while none in pandemic H1N1/2009 influenza A (pH1N1) viruses. We investigated the effect of the two glycosylation sites on viral virulence and pathogenicity in mice using recombinant pH1N1. The H1N1/144 and H1N1/177 mutants which gained potential glycosylation sites Asn142 and Asn177 on HA respectively were generated from A/Mexico/4486/2009(H1N1) by site-directed mutagenesis and reverse genetics, the same as the H1N1/144+177 gained both glycosylation sites Asn142 and Asn177. The biological characteristics and antigenicity of the mutants were compared with wild-type pH1N1. The virulence and pathogenicity of recombinants were also detected in mice. Our results showed that HA antigenicity and viral affinity for receptor may change with introduction of the glycosylation sites. Compared with wild-type pH1N1, the mutant H1N1/177 displayed an equivalent virus titer in chicken embryos and mice, and increased virulence and pathogenicity in mice. The H1N1/144 displayed the highest virus titer in mice lung. However, the H1N1/144+177 displayed the most serious alveolar inflammation and pathogenicity in infected mice. The introduction of the glycosylation sites Asn144 and Asn177 resulted in the enhancement on virulence and pathogenicity of pH1N1 in mice, and was also associated with the change of HA antigenicity and the viral affinity for receptor.

## Introduction

Influenza A viruses are responsible for both seasonal epidemics and occasional pandemics in human. The emergence of new influenza virus strains to which the general population has little or no immunity, such as the pandemic H1N1/2009 influenza A (pH1N1) viruses, can easily transmit from one person to another and rapidly spread across the globe [1]. Under the pressure of the host's immune system, the pandemic viruses need to change its antigenic structure (called antigenic drift) so as to escape from the defenses. Such pressure and drift could be why influenza immunity is not always neutralizing, as minor variations to the virus render it “unknown” to the hosts' adaptive immune response [2], [3].

Glycans on the hemagglutinin (HA) of infl uenza A virus attach through N-glycosidic linkages to asparagine residues (Asn) of the conserved glycosylation site motif Asn-X-Ser/Thr, in which X may represent any amino acid except proline [4], [5]. HA serves as the major target for neutralizing antibodies, and glycans expressed on the head of HA are likely to shield or modify antigenic sites [6]. Glycosylation of HA can affect the host specificity, infectivity and virulence of an influenza strain either directly, by changing the biological properties of HA [7] or other mechanisms such as shielding antigenic regions of the protein [8]–[11], impeding the activation of the protein precursor HA_0_ via its cleavage into the disulfide-linked subunits HA_1_ and HA_2_ [12]–[14], or attenuating receptor binding capability [15]–[19]. It has been reported that removal of both Asn165 and Asn246 of H3N2 influenza viruses led to a further increase in virulence, characterized by enhanced virus replication, pulmonary inflammation and vascular leak [20]. Addition of glycosylation sites in PR8 HA was sufficient to attenuate disease and removal of glycans from Brazil HA resulted in severe disease and death [21]. Additionally, glycosylation at the 158N site and the receptor binding preference of the VN04 (H5N1) ca vaccine virus affected virus antigenicity and caused poor replication in the host [22].

Some glycosylation sites are highly conserved, while the location and number of the other sites vary between viruses [16], [23]. As it reported that the seasonal H1N1 viruses possessed more N-glycosylation sites in their HA sequences than the 1918 H1N1 strain (A/South Carolina/1/18) and it was associated with the host adaptation of the viruses [24]–[26].

Based on the sequence comparing, we found that two glycosylation sites at Asn142 and Asn177 on the HA in most pre-2009 human seasonal influenza A H1N1 viruses, but not in 2009 pH1N1 viruses (T144D, N177K). Here we used site-directed mutagenesis to add potential glycosylation sites (Asn142 and Asn177) into the HA of A/Mexico/4486/2009(H1N1). One gained site Asn142 (H1N1/144), one gained site Asn177 (H1N1/177) and another both sites Asn142 and Asn177 (H1N1/144+177), to compare the biological property with the wild virus (H1N1/WT). The information here provides additional understanding of the pandemic 2009 H1N1 strains pathogenicity and virulence.

## Materials and Methods

### Viruses, cells and animals

Six weeks old female BALB/c mice were performed according to protocols approved by the Hubei Provincial Animal Care and Use Committee (approval number: SYXK 2010–0029). Influenza A virus used in this study were A/Mexico/4486/2009(H1N1), a pandemic (H1N1) 2009 virus. The GenBank accession numbers of the genome are GQ149617-24 and the HA is GQ149623. Human embryonic kidney (293T) cells and Madin-Darby canine kidney (MDCK) cells were obtained from the American Type Cluture Collection (ATCC) and cultured in DMEM supplemented with 10% fetal bovine serum, 1% penicillin-streptomycin. Cultures were incubated at 37°C with 5% CO_2_.

### Generation of glycosylation mutant viruses using reverse genetics (RG)

Reassortant IAV used in this study were generated by eight-plasmid reverse genetics as previously described [27]. To add N-glycosylation sites to HA, nucleic acid mutations were performed to facilitate amino acid substitutions that created glycosylation motifs (Asn-X-Ser/Thr) at sites Asn142 (D144T) and Asn177 (K177N). Site-directed mutagenesis was performed using Pfu DNA polymerase (Stratagene).

### Examination of HA glycosylation by Western blot

To confirm whether glycosylation motifs at sites Asn142 (D144T) and Asn177 (K177N) were present in the HA protein of pH1N1, Western blotting was performed to examine the mobility change of the HA protein on a polyacrylamide gel. Each virus was concentrated by ultracentrifugation and viral proteins were electrophoresed on a Novex® 10% Tris-glycine gel (Invitrogen). The electrophoresed proteins on the gel were transferred to a nitrocellulose membrane, and the membranes were blocked in 1% fat-free milk before incubation with monoclonal antibodies specific against HA of pH1N1/WT and then incubated with goat anti-mouse antibody. Protein bands were detected with ECL (Amersham) by DNR Bio Imaging System.

### 50% egg infectious dose (EID_50_) in chicken embryos and viral growth kinetics

The virulence of the H1N1 wild-type and the H1N1/144, H1N1/177, H1N1/144+177 were determined by the EID_50_ in embryonated SPF chicken eggs. To analyze viral replication, a comparison of viral growth kinetics for four viruses was undertaken in embryonated SPF chicken eggs at 37°C. The viral titers in the allantoic fluid of infected eggs were detected at 24, 48, 72 and 96 h after infection. The EID_50_ was calculated by the method of Reed and Muench [28].

### Virus elution assay

The ability of HA bind on erythrocytes was assessed as described previously [29]. Briefly, 50µl of twofold dilutions of virus containing HA titers of 1∶32 was incubated with 50µl of 0.5% chicken erythrocytes in microtiter plates at 4°C for 1 h. The microtiter plates were then stored at 37°C, and the reduction of HA titers was recorded periodically for 4 h. Calcium saline (6.8 mM CaCl_2_-154 mM NaCl in 20 mM borate buffer, pH 7.2) was used as a diluent.

### Hemagglutination Inhibition of the Mutants to Different Monoclonal Antibodies

The monoclonal antibodies 5D_5_, 4D_7_, 4E_1_, 2B_3_, 3G_12_, 1C_9_, 4B_12_, 2C_5_, 2H_7_ and 5F_7_ were specific against HA of pH1N1/WT and were used for the pH1N1 mutants in HA inhibition (HAI). The HAI procedure was performed essentially as described [30]. The HAI titer was expressed as the reciprocal of the last serum dilution achieving complete inhibition of agglutination.

### Microneutralization assay

Serial two-fold dilutions of monoclonal antibodies were incubated with an equal volume of the indicated viruses at a concentration of 100 50% tissue culture infectious dose (TCID_50_)/ml in a 96-well U-bottom plate for 60 min at 33°C. The virus-antibody mixture was transferred to monolayers of MDCK cells and incubated at 37°C for 4 days. The neutralizing antibody titers were defined as the reciprocal of the highest antibody dilution that completely neutralized the appropriate virus as defined by the absence of CPE on day 4 post infection.

### Infection of mice

Groups of eight 6-week-old female BALB/c mice were anesthetized with methoxyflurane and 50µL of infectious viruses diluted in PBS were inoculated intranasal. For comparison of morbidity (measured by weight loss), mortality, and virus distribution in lung, additional mice were infected with inoculating doses of 10^3^ EID_50_ of the viruses. Mice were observed daily for 14 days for weight loss and mortality. The virus titer in the lung was expressed in relative NP gene expression on days 2, 5, 7, and 9 after infection, 5 mice from each group were sacrificed, and lung samples were harvested, and total RNA was extracted using TRIzol (Invitrogen). The relative NP genes were detected by real-time PCR.

### Measurement of IL-1, IL-10, MCP-1, TNF-α,IFN-γ mRNA by real-time RT-PCR

5 mice from each group were sacrificed on days 2, 5, 7, and 9 after infection, then lung samples were harvested, and total RNA was extracted using TRIzol (Invitrogen). Complementary DNA (cDNA) of IL-1, IL-10, MCP-1, TNF-α, IFN-γ were synthesized with the Reverse Transcriptase XL (TaKaRa) and oligo dT primer (Toyobo). Each cDNA sample was used as a template for a real-time PCR amplification with reaction mixture containing SYBR Green I (Toyobo), and all forward and reverse primers were showed in table 1. GAPDH was used for a control. Virus titers in the tissue homogenates were determined by real-time RT-PCR. The fold-changes were calculated as previously described by Livak and Schmittgen [31].

**Table 1:** Primers for real-time RCR.

Cytokine	Primers	
	Forward	Backward
IL-1	5′-CACCTGGTACATCAGCACCTCAC-3′	5′-CATCAGAAACAGTCCAGCCCATAC-3′
IL-10	5′-GGT TGCCAAGCC TTATCGGA-3′	5′-ACCTGCTCCACTGCCTTGCT -3′
MCP-1	5′-CCAGCAAGATGATCCCAATGA-3′	5′-CAGTTGGTTCCGATCCAGGTT -3′
TNF-α	5′-CGATGAGGTCAATCTGCCCA-3′	5′-CCAGGTCACTGTCCCAGCATC-3′
IFN-γ	5′-TGGTGGTGATGTCTACACTCCG-3′	5′-CGAGTTATTTGTCATTCGGGTGT-3′
NP	5′-CAGGAAACGCTGAGATTGAA-3′	5′-TGGGTTTTCATTTGGTCTCA-3′
GAPDH	5′-GAAGGGCATCTTGGGCTCACT-3′	5′-GGTGGGTGGTCCAGGGTTTCTTA-3′

**NOTE**. GAPDH, glyceraldehyde 3-phosphate dehydrogenase; IFN, interferon; IL, interleukin; MCP, monocyte chemotactic protein; TNF, tumor necrosis factor.

### Pulmonary histopathology of infected mice

On days 2, 5, 7 and 9 after infection, 3 mice from each group were sacrificed, and lung samples were removed and immersed in 4% formaldehyde for a minimum of 24 h. After fixation of the lung tissue and processing in paraffin wax, sections (4µm thick) were prepared and stained with H&E as described previously [32].

## Results

### Comparison of glycosylation sites on HA of pandemic H1N1 influenza viruses with seasonal and swine H1N1 influenza viruses

HA sequences of 885 pre-2009 human seasonal influenza H1N1 viruses were obtained from Influenza Virus Database (www.ncbi.nlm.nih.gov/genomes/FLU/) and were searched for glycosylation consensus sequence sites (142 and 177). Glycosylation sites were identified in 754 out of 885 sequences at residue 142, and 788 out of 885 sequences at residue 177.

However, out of >2000 human pandemic H1N1 strains examined from the Influenza Virus Database, there is no glycosylation site present at residue 142 or residue 177. Because the HA of pandemic H1N1 is a swine-origin HA, we also examined HAs of H1N1 swine isolates in North American and China for glycosylation sites at these locations. Very few glycosylation sequences were observed in H1N1 swine isolates at residue 142 and 177 (Table 2).

**Table 2:** The glycosylation sites on HA in different types of A/H1N1 influenza viruses.

Pandemic 2009 H1N1	144	177	Seasonal H1N1	144	177	Swine H1N1 in North America	144	177	Swine H1N1 in China	144	177
A/Mexico/4482/2009	D	K	A/Mexico/UASLP-009/2008	T	N	A/swine/Iowa/H04YS2/2004	D	K	A/swine/Liaoning/32/2006	D	K
A/California/04/2009	D	K	A/California/11/2008	T	N	A/swine/MN/48683/2002	D	K	A/swine/Shandong/692/2008	D	K
A/Mexico/4486/2009	D	K	**A/California/06/2008**	T	**K**	A/swine/Minnesota/01358/2006	D	K	A/swine/Zhejiang/1/2007	D	K
A/New York/1682/2009	D	K	A/California/05/2007	T	N	A/swine/Ontario/57561/03	E	K	A/swine/Beijing/26/2008	D	K
A/England/195/2009	D	K	A/California/10/2006	T	N	A/swine/Saskatchewan/18789/02	E	K	A/swine/Fujian/204/2007	D	K
A/England/886/2009	D	K	A/Guangzhou/665/2006	T	N	A/swine/North Carolina/7386/2004	D	K	A/swine/Guangdong/628/2006	E	K

**NOTE.** The amino acid of potential glycosylation sites was T (144) and N (177) on HA.

### The generation and characterization of influenza viruses containing different N-glycosylation sites on HA

**Figure 1:**
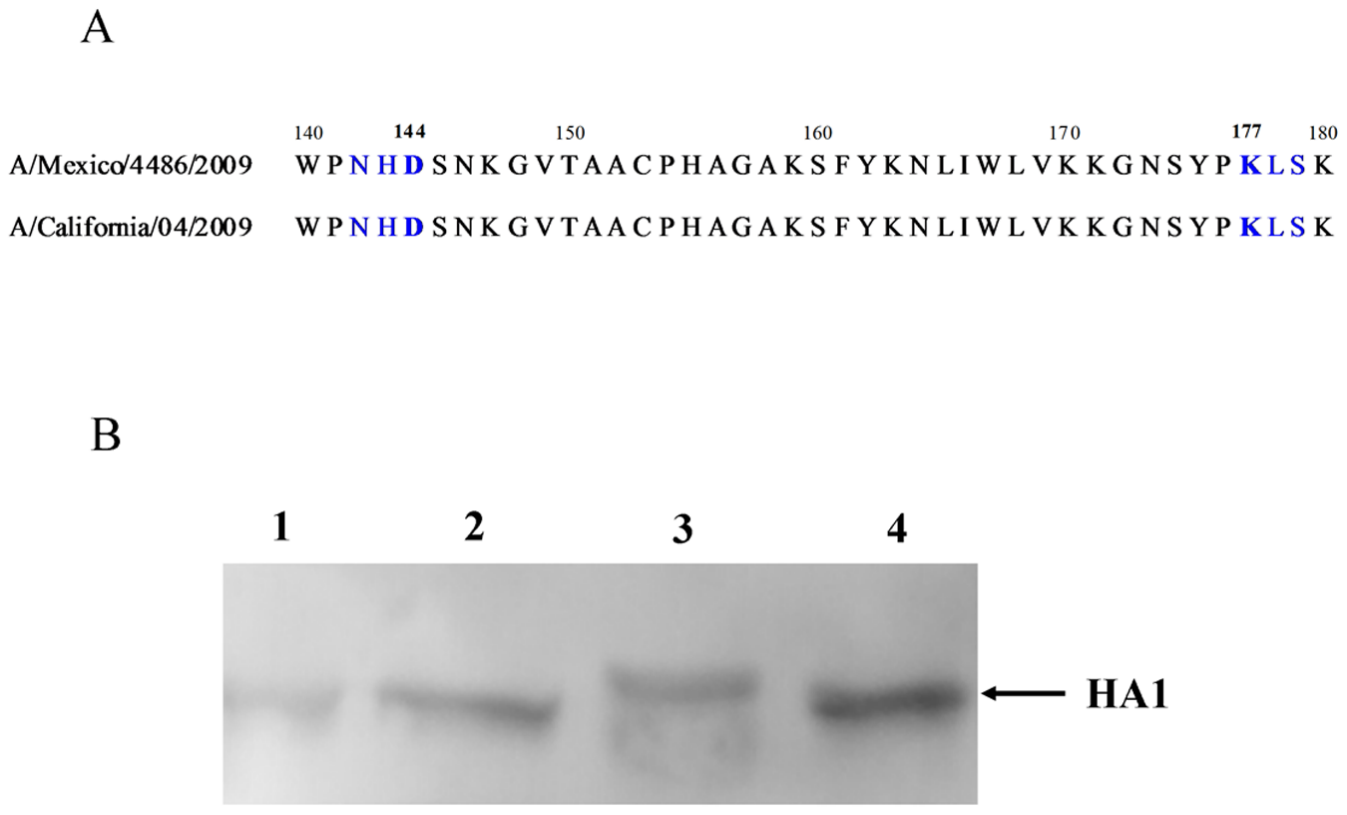
HA sequence alignment and examination of glycosylation. (A) The HA protein sequence alignment of pandemic H1N1/2009 virus. (B) Examination of glycosylation of the H1N1 HA proteins by Western blotting. 1: H1N1/144, 2: H1N1/177, 3: H1N1/144+177 and 4: H1N1/WT.

With A/Mexico/4486/2009(H1N1) strain as backbone, we generated two single mutants (144, 177) and one double mutant (144+177). All the recombinant viruses were sequenced, and no additional mutations were introduced. To confirm that the Asn142 (D144T) and Asn177 (K177N) glycosylation sites in HAs of the mutants were indeed utilized, the HAs of H1N1/144, H1N1/177, H1N1/144+177 and H1N1/WT viruses were analyzed by Western blot using an H1 HA-specific antibody (Fig. 1B). As expected, the HA of the H1N1/144+177 virus with the 144T-177N sequence in the HA1 migrated slower than the H1N1/WT virus with 144D-177K. However, no obvious difference was observed between single site mutant virus and wild-type virus.

As shown in Table 3, H1N1/144 had a highest EID_50_ (10^7^), which was approximately 2-fold higher than H1N1/144+177 (10^6.7^). The EID_50_ of H1N1/177 (10^5.5^) and H1N1/WT (10^4.7^) were 32-fold to 200-fold lower than H1N1/144. For virus titers on chicken embryos (HA titers), the mutants H1N1/144 (2^8^) and H1N1/144+177 (2^7^) showed a higher level than did H1N1/177 (2^5^) and H1N1/WT (2^5^).

**Table 3:** In Vitro Characteristics of the Recombinant Viruses.

Virus	Embryonated chicken eggs		Pathogenicity in MDCK
	EID_50_ (log_10_)	HA titer (log_2_)	TCID_50_/100µl (log_10_)
H1N1/144	7.0	8	5.2
H1N1/177	5.5	5	3.5
H1N1/144+177	6.7	7	5.5
H1N1/WT	4.7	5	5.0

**Note.** EID_50_, 50% egg infectious dose; HA, hemaglutination; TCID_50_, 50% MDCK cell infectious dose.

The HI titers of the monoclonal antibodies (specific to HA of pH1N1/WT) with H1N1/177 were similar to H1N1/WT, whereas the reaction of H1N1/144 with 2H_7_ was undetectable and the HI titers of 5D_5_, 4E_1_, 3G_12_, 2C_5_ and 2H_7_ with H1N1/144+177 were significantly lower than H1N1/WT (Table 4). And the results of microneutralization assay are consistent with the results from HI assay (Table 4). It indicated that glycosylation site (Asn 144) on HA affect the antigenicity of mutants, and one of the antigen sites may change.

**Table 4:** Effect of variations at four HAs on reactivity with monoclonal antibodies.

Virus	HAI (NT) titers with monoclonal antibodies^1^									
	5D_5_	4D_7_	4E_1_	2B_3_	3G_12_	1C_9_	4B_12_	2C_5_	2H_7_	5F_7_
H1N1/144	2^18^	2^16^	2^12^(3698)	2^10^	2^16^(22594)	2^15^	2^10^	2^17^(5382)	0(10)	2^18^
H1N1/177	2^15^	2^15^	2^13^(3404)	2^12^	2^16^(5093)	2^16^	2^16^	2^13^(1402)	2^16^(4988)	2^18^
H1N1/144+177	**2^9^**	2^16^	**2^4^**(57)	2^11^	**2^7^**(1100)	2^17^	2^13^	2^8^(1300)	2^6^(80)	2^16^
H1N1/WT	2^18^	2^16^	2^16^(6267)	2^11^	2^16^(21527)	2^17^	2^13^	2^17^(6180)	2^16^(3672)	2^19^

**Note.** ^1^ The monoclonal antibodies(5D_5_,4D_7_,4E_1_,2B_3_,3G_12_,1C_9_,4B_12_,2C_5_,2H_7_,5F_7_) were specific against HA of pH1N1/WT. HI tests were performed by standard methods using chicken red blood cells. (NT), microneutralization assay.

**Figure 2:**
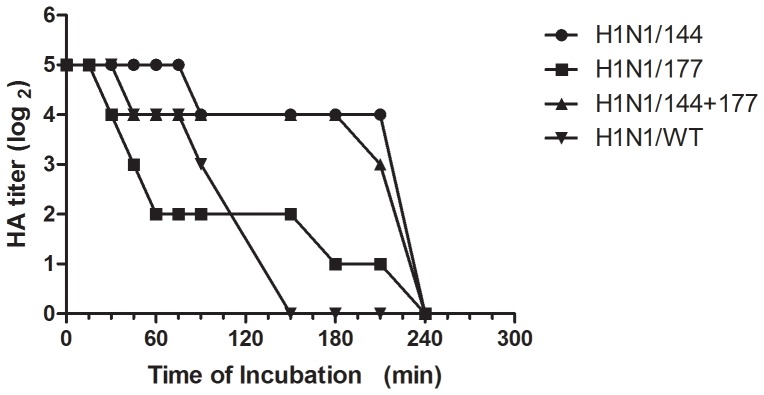
Virus elution from erythrocytes. Two-fold dilutions of virus containing the HA titers of 1∶32 (2^5^) were incubated with equal volume of 0.5% chicken erythrocytes in microtiter plates at 4°C for 1 h. The microtiter plates were then stored at 37°C, and the reduction of HA titers was recorded periodically for 240 min.

We compared the ability of the mutant HAs to bind and elute from chicken erythrocytes, first by performing hemagglutination at 4°C followed by incubation at 37°C. Because the NAs were the same, comparison of the elution properties revealed the correlation between different N-glycosylation sites and the ability of the HA to bind erythrocytes (Fig. 2). The H1N1/WT was released completely from erythrocytes by 2.5 h of incubation at 37°C, whereas longer times were required for release of H1N1/177, H1N144+177, H1N1/144. The H1N144+177 and H1N1/144 displayed a slower release from erythrocytes than did the H1N1/177. These showed the HA binding activity to erythrocytes was increased by introducing the Asn142 or Asn177 in HA.

### Viral growth kinetics

**Figure 3:**
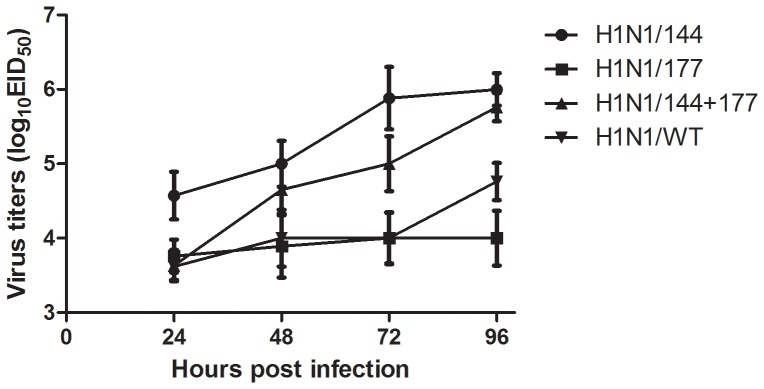
Characterization of viral growth in embryonated SPF chicken eggs. The eggs were infected with each of the viruses and were maintained at 37°C. The viral titers in the allantoic fluid of infected eggs were examined at 24, 48, 72, 96 h after infection by EID_50_. Titers were expressed as log_10_ EID_50_.

Growth of viruses was examined in embryonated SPF chicken eggs. Viral replication kinetics were determined at 24, 48, 72 and 96 h after infection by EID_50_ and HA titer. The H1N1/144 mutant replicated to the highest titers in eggs, with H1N1/144-177 reaching slightly lower virus titers during the 96 h post infection compared with H1N1/WT and H1N1/177. The EID_50_ of H1N1/177 mutant equaled to the H1N1/WT during the first 72 h post infection, and then it was slightly lower than H1N1/WT in the next 24 h (Fig. 3). The viruses reached the highest HA titer after 72 h; H1N1/144 (2^8^) was higher than H1N1/144+177 (2^7^), then H1N1/177 and H1N1/WT were lower (both 2^5^).

### In vivo characterization of recombinant viruses in the mouse model

**Figure 4:**
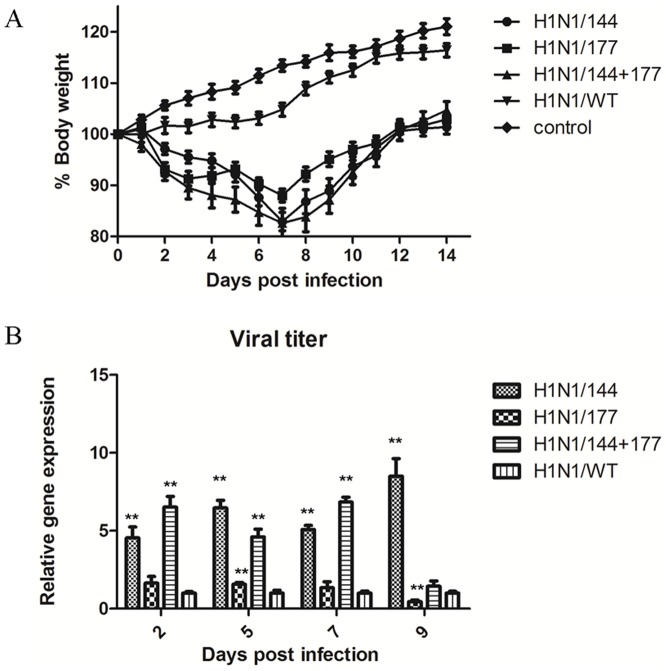
Weight loss and lung virus titres in infected mice. (A) Weight loss of mice inoculated with strains having different N-glycosylation sites on HA. Six-week-old BALB/c mice were inoculated intranasal with 10^3^ 50% egg infectious dose (EID_50_) of the recombinant influenza viruses, with 8 mice per group. P<0.05 between H1N1/144+177 and H1N1/WT on 3, 4 days post infection, P<0.01 between H1N1/144+177 and H1N1/WT on 5 days post infection, P<0.05 between H1N1/177 and H1N1/WT on 3–12 days post infection, P<0.01 between H1N1/177 and H1N1/WT on 13, 14 days post infection, P<0.01 between H1N1/144 or H1N1/144+177 and H1N1/WT on 6–14 days post infection. (B) Viral titres in lung of infected mice were indicated by the expression of NP gene. The statistical analysis was performed using Student's t test. *, P<0.05; **, P<0.01 between the recombinant mutants and H1N1/WT viral titer.

Four groups of mice were inoculated intranasal with 1000 EID_50_ of the recombinant viruses and another group was inoculated intranasal with 50µL PBS as the control. The body weight of each mouse was measured daily until 14 days after infection. Mice infected with the recombinant mutants showed more signs of illness compared with the WT group. All mice infected with any of the recombinant viruses experienced body weight loss except the mice infected with wild-type viruses (Fig. 4A). The body weight got loss at 2 days post infection, with H1N1/144+177 and H1N1/144 causing 18% weight loss on days 7 after infection. The mutant H1N1/177 caused lower weight loss than did H1N1/144+177 and H1N1/144, and 12% weight loss on days 7 after infection. H1N1/144, H1N1/177, H1N1/144+177, H1N1/WT did not cause any death in mice and all mice recovered from infection.

The virus titers in the lung of infected mice were also compared by real-time PCR. The H1N1/144 and H1N1/144+177 showed significantly higher titers than H1N1/177 and H1N1/WT on days 2, 5, 7, and 9 after infection, and H1N1/177 was slightly higher than H1N1/WT on days 9 post infection (Fig. 4B).

### Four virus strains induced the expression of antiviral and proinflammatory cytokines in murine lung

**Figure 5:**
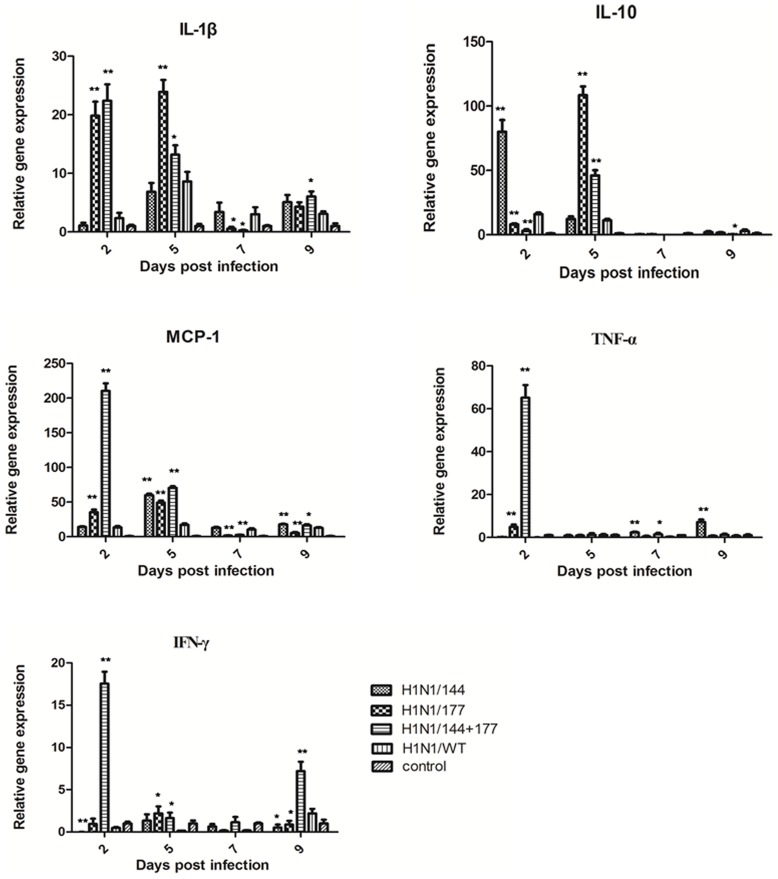
The levels of antiviral and proinflammatory cytokines in lung of infected mice. Determination of IL-1, IL-10, MCP-1, TNF-α and IFN-γ levels in H1N1/144, H1N1/177, H1N1/144+177 or H1N1/WT-infected mouse lungs at 2, 5, 7, 9 d.p.i. The statistical analysis was performed using Student's t test. *, P<0.05; **, P<0.01 between the recombinant mutants and H1N1/WT.

To determine if there was a correlation between severe disease and proinflammatory cytokine production, the murine lungs were infected with the mutants. Real-time PCR was used to determine the difference in proinflammatory cytokine production following infection with the four H1N1 viruses in the mouse lung, including IL-1, IL-10, MCP-1, TNF-α, IFN-γ (Fig. 5). The H1N1/177 and H1N1/144+177 induced relatively higher IL-1 mRNA than did H1N1/WT and the control. The expression of MCP-1, TNF-α, and IFN-γ genes were significantly higher in mice infected with the H1N1/144+177. The production of IL-10 induced by the H1N1/144 and H1N1/177 was higher than in other viruses.

### Pulmonary histopathology following infection of mice with glycosylation mutants

**Figure 6:**
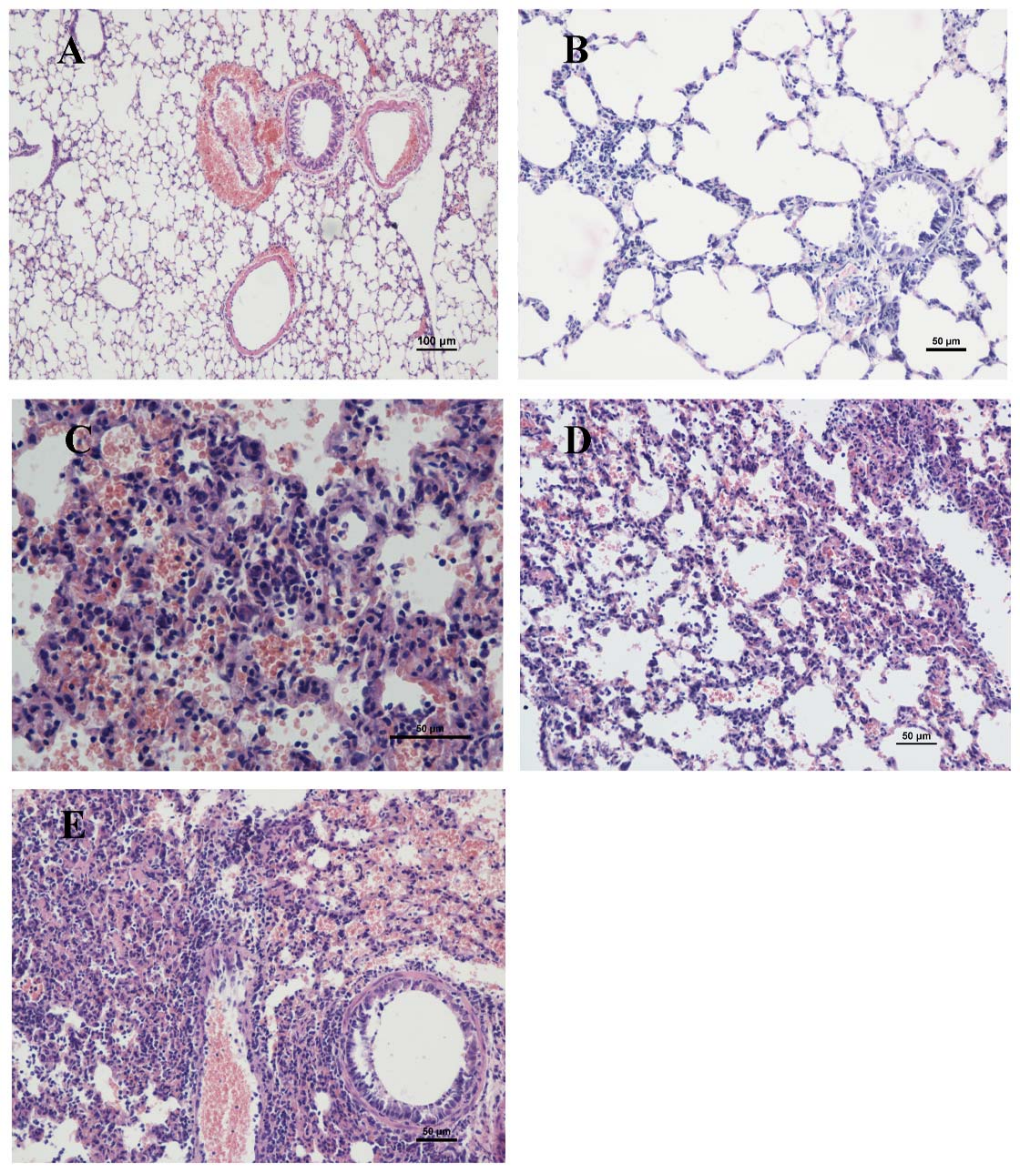
Histopathologic changes of lung from H1N1 virus-infected mice. Photomicrographs of hematoxylin-and-eosin strained lung sections from mice at 7 days p.i. are shown. Mice were infected with mock (A), H1N1/WT (B), H1N1/144 (C), H1N1/177 (D), H1N1/144+177 (E).

We determined lung histological pathology from mice infected with 10^3^ EID_50_ H1N1/144, H1N1/177, H1N1/144+177 or H1N1/WT at day 7 post infection (Fig. 6). Histologically, all mutants' infections produced lesions typical of influenza A virus infections: bronchiolitis with accompanying necrosis of respiratory epithelium and associated neutrophilic to histiocytic alveolitis. However, the severity and character of necrosis and inflammation varied with individual virus strains. The most severe lesions observed were with H1N1/144+177, which produced mild to severe necrosis of bronchiolar epithelium with inflammatory cell infiltrate, pulmonary edema, lung parenchyma and pulmonary congestion. Infection with H1N1/144 and H1N1/177 viruses resulted in lesions in the lung varying from mild to moderate bronchiolitis with occasional necrosis of bronchiolar epithelium and mild to moderate peribronchiolar alveolitis. The mildest lesions were seen with H1N1/WT viruses which produced mild bronchiolitis with minimal to no respiratory epithelial necrosis and only mild histiocytic alveolitis associated with terminal bronchioles. In summary, the mutants were capable of causing more serious histological lung pathology than H1N1/WT.

### Structural locations of mutations

**Figure 7:**
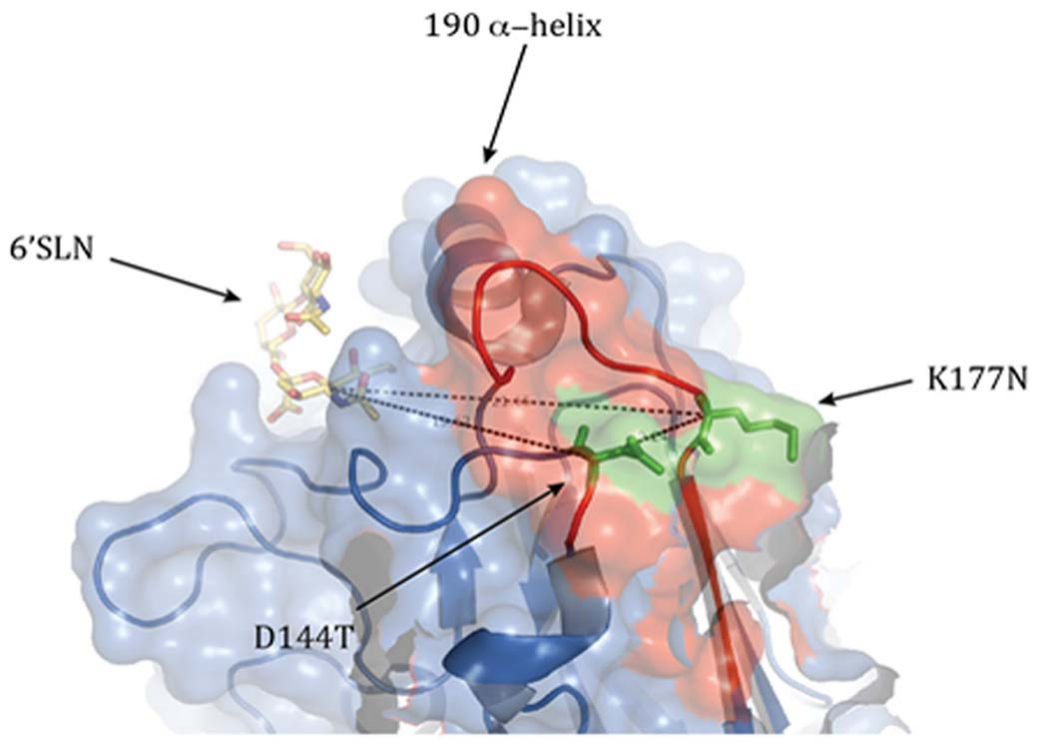
Structural locations of mutations found in pseudorevertant viruses. The antigenic site Sa is colored red. 144^th^ and 177^th^ sialic acids modeled as green stick structure. Images are derived from the crystal structure of the hemagglutinin of A/California/04/2009 H1N1 virus. Images were generated using the MacPyMol software and the solved HA structure (ID 3LZG; RCSB Protein Data Bank).

Locations of 144 and 177 mutations are in the globular head near the vicinity of the receptor binding site 6′ SLN and the distance is respectively 19.62Å, 21.65Å. Residue 144 is very close to the antigenic site Ca_2_ and residue 177 is located in the antigenic site Sa (Fig. 7). The presence of a glycosylation site here would shade the antigenic region Ca_2_ or Sa from antibody binding.

## Discussion

Some of the potential glycosylation sites, such as 28, 40, 104, 304, 498 and 557 on HA, are highly conserved in all H1N1 strains isolated from various animals and human. While other sites at residues 142, 172, 177 and 179 on the top of the HA head, and 71 and 286 on the side of the HA head only appeared during certain evolutionary periods for the human seasonal influenza H1N1 viruses [33]. Analysis of H1 sequences indicate that glycosylation on the receptor binding domain of HA is present in most pre-2009 seasonal IAV but is absent in all 2009 pandemic virus strains. It has been proven that the acquisition of potential glycosylation sites is one of the effective ways for influenza viruses to escape positive selective pressures from the hosts [7], [34]. Previous studies showed that three potential N-linked glycosylation sites (aa131, aa158 and aa169) were frequently present within or near the receptor binding site of the H5 HA and the glycosylation at 158 was reported to influence the antigenicity of H5N1 viruses isolated in Hong Kong in 1997 [16]. Additionally, sequence analysis of circulating H1 influenza viruses confirmed the in vivo relevance of the findings: natural occurrence of glycosylation at residue 131 is always accompanied by a compensatory mutation known to increase HA receptor avidity [11]. In this study, we systematically defined the role that particular glycosylation sites on pandemic H1N1/2009 IAV played in modulating sensitivity to pathogenesis and virulence in mice.

The H1N1/144 displayed the highest virus titer in lung in mice than the H1N1/144+177, H1N1/177 and H1N1/WT. However, the H1N1/144+177 exhibited the most serious virulence and pathogenicity in infected mice. None of the mutants caused death in mice. The H1N1/177 exhibited an equivalent virus titer in chicken embryos and mice, and increased virulence and pathogenicity in mice.

Previous studies showed deletion of Asn144 on HA from seasonal influenza Brazil/H1N1 reduced sensitivity to mouse bronchoalveolar lavage (BAL) and increased virulence in mice. Moreover, simultaneous addition or deletion of Asn104 and Asn144 from PR8/H1N1 or Brazil/H1N1 HA, respectively, led to marked changes in sensitivity to mouse BAL and virulence when compared with loss of either site alone. Single-site deletion of Asn177 reduced sensitivity to mouse BAL and increased virulence in mice [21]. In contrast, in pH1N1, introduction of a glycosylation site at Asn142 and Asn177 on the HA of pandemic H1N1/2009 resulted in increased pathogenesis and virulence in mice. The difference may be caused by different strains which differ greatly in their HA sequences.

The times required for elution from chicken erythrocytes were longer than H1N1/WT, which showed increased binding affinity on HA for sialic acid. This indicated the addition of glycosylation sites on HA may affect the viral affinity for a particular receptor. And the HI titers to 5D_5_, 4E_1_, 3G_12_, 2C_5_ and 2H_7_ with H1N1/144+177 were significantly lower than H1N1/WT. Significantly, there was no HI titers of 2H_7_ H1 monoclonal antibodies responded with H1N1/144. These results indicated that the addition of glycosylation sites may change the antigen sites of the mutants, which led to that the ability of these antibodies to neutralize the virus was largely attenuated.

Previous studies demonstrated that glycosylation sites in HA receptor binding domain (RBD) were important in allowing evasion of antibody neutralization in seasonal strains and introduction of glycosylation sites eliminated the viral ability to bind neutralizing antibodies, suggesting that glycan shielding from antibodies was a mechanism by which seasonal influenza viruses evolved after the emergence of a viral pandemic strain [24]. In this study, the pandemic H1N1 strain acquired glycosylation sites on the RBD that can effectively mask antigenic regions from recognition by antibodies.

Similar to findings from recent studies, the addition of Asn142 and Asn177 to pandemic H1N1 HA trimers was associated with reduced sensitivity to neutralizing Abs raised to WT pandemic HA [24]. All these suggested that glycan shielding on HA may be an important mechanism by which pandemic viruses entering the human population evolve into seasonal influenza virus strains. This also explained why the pandemic virus was antigenically distinct from seasonal influenza viruses, and the majority of human population lacked immunity against this virus.

Influenza virus infection results in the production of several chemokines, including monocyte chemotactic protein 1 (MCP-1), which plays a role in the recruitment of leukocytes to sites of infection. A recent study described the increased production of several cytokines in mice infected with the 2009 H1N1 virus CA/4 compared with a seasonal H1N1 virus [35]. Currently, there is little information on the proinflammatory response induced by 2009 H1N1 viruses gain glycosylation sites and its relevance to disease severity in mice. In this study, levels of IL-1, IL-10 and MCP-1 following the glycans mutant 2009 H1N1 virus infection were significantly higher compared with a less virulent wild H1N1 virus, similar to the results in the study by Itoh *et al*., which demonstrated elevated levels of cytokines from CA/4 virus compared with a less virulent seasonal H1N1 virus [35]. Our studies showed that levels of IL-1, IL-10, MCP-1, TNF-α and IFN-γ following H1N1/144+177 infection were significantly higher compared with H1N1/WT virus, and H1N1/144 and H1N1/177 did not induce all of these cytokine up-regulation but two or three. The cytokine production profiles with the influenza viruses obtained here correlated with the viral replication levels in the lung of infected mice.

Previous studies suggested that, in fatal human cases of the H5N1 infections, a virus-induced “cytokine storm” contributed to the severity of the disease [36]. Moreover, in human macrophages the highly pathogenic H5N1 virus induced very strong cytokine and IFN responses [37]. Strong cytokine responses induced by the mutant H1N1/144+177 are associated with the viral increased virulence and may be a reason to severe lung damage in mice.

The previous results showed that glycosylation sites migrations in human influenza viruses affected the function by several mechanisms. They can stabilize the polymeric structures, regulate the receptor binding, more effectively mask the antigenic sites, more effectively protect the enzymatic cleavage sites of neuraminidase (NA) and catalytic activities and balance the binding activity of HA with the release activity of NA [2]. The findings of this study have showed that the pandemic H1N1 strain can effectively mask antigenic regions from recognition by antibodies according to acquired glycosylation sites on HA. Similar findings occurred with H2N2, H3N2, and H5N1 viruses, where glycosylation of HA was shown to inhibit recognition by antibodies [8], [10], [38], [39] and was associated with their antigenic drift [6].

Together, our data showed the importance of 144 and 177 sites of N-linked glycosylation on the pandemic H1N1 HA on receptor binding and the antigenic sites, also on viral replication and virulence in embryonated SPF chicken eggs and mice. The data in this study suggested that an effective escape mode for the pandemic H1N1/2009 virus to the present vaccine would be conferred by acquisition of glycosylation sites in the HA-RBD region, which will help to preemptively develop vaccine strains to prevent the emergence of HA-RBD glycosylated viruses, thus constraining its evolution into a seasonal influenza and limiting its further spread. Given the possible production of the novel viruses of potential threat to public health, we should emphasize influenza surveillance and establishment of the genetic basis of the viral genome for rapidly identifying such mutant events.
